# Prion-like Domains in Spike Protein of SARS-CoV-2 Differ across Its Variants and Enable Changes in Affinity to ACE2

**DOI:** 10.3390/microorganisms10020280

**Published:** 2022-01-25

**Authors:** George Tetz, Victor Tetz

**Affiliations:** 1 Department of Proteome Research, Human Microbiology Institute, New York, NY 10013, USA; info@hmi-us.com; 2 Department for Proteome Research, Tetz Laboratories, New York, NY 10013, USA

**Keywords:** COVID-19, SARS-CoV-2, variants, prion-like domains, PrD, ACE2, Delta variant, Omicron variant

## Abstract

Currently, the world is struggling with the coronavirus disease 2019 (COVID-19) pandemic, caused by severe acute respiratory syndrome coronavirus 2 (SARS-CoV-2). Prions are proteins that possess a unique conformational conversion, with the ability to rapidly shift between multiple conformations due to residue hydrophobicity and net sequence charge, and viral prion-like proteins are known as potential regulators of viral infections. However, the prion-like domains (PrD) in the SARS-CoV-2 proteome have not been analyzed. In this in silico study, using the PLAAC algorithm, we identified the presence of prion-like domains in the SARS-CoV-2 spike protein. Compared with other viruses, a striking difference was observed in the distribution of prion-like domains in the spike protein since SARS-CoV-2 is the only coronavirus with a prion-like domain found in the receptor-binding domain of the S1 region of the spike protein. The presence and unique distribution of prion-like domains in the SARS-CoV-2 receptor-binding domains of the spike protein are particularly interesting since although the SARS-CoV-2 and SARS-CoV S proteins share the same host cell receptor, angiotensin-converting enzyme 2 (ACE2), SARS-CoV-2 demonstrates a 10- to 20-fold higher affinity for ACE2. We identified prion-like domains in the α1 helix of the ACE2 receptor that interact with the viral receptor-binding domain of SARS-CoV-2. Finally, we found substantial differences in the prion-like domain of the S1 region of the spike protein across emerging variants including Omicron (B.1.1.529). Taken together, the present findings indicate that the identified PrDs in the SARS-CoV-2 receptor-binding domain (RBD) and ACE2 region that interact with RBD play important functional roles in viral adhesion and entry.

## 1. Introduction

The world is struggling with the pandemic caused by a novel coronavirus (now named severe acute respiratory syndrome-2 or SARS-CoV-2, causing coronavirus disease 2019 (COVID-19)) that has expanded from Wuhan throughout China and the wider world [1]. By 12 December 2021, there were over 259 million confirmed cases of the virus worldwide and it had contributed to over 5.2 million deaths (https://www.worldometers.info/coronavirus/, accessed on 10 January 2022).

SARS-CoV-2 is a new member of the Betacoronavirus (β-CoV) genus of large, enveloped, single-stranded RNA viruses [2]. This genus not only includes viruses that cause deadly human infections such as severe acute respiratory syndrome (SARS) and Middle East respiratory syndrome (MERS) but also encompasses viruses that cause non-life-threatening common colds, including human coronavirus OC43 (HCoV-OC43) and human coronavirus HKU1 (HCoV-HKU1) [3]. Although these viruses predominantly infect lung epithelial cells, the clinical severity and pathogenesis of the infections they cause vary between different coronaviruses [4]. While severe pneumonia and pulmonary fibrosis are fundamental to the pathogenesis of COVID-19, SARS and MERS, these symptoms are not typical of infections caused by HCoV-OC43 and HCoV-HKU1 [5,6].

Like other β-CoVs, the genome of the novel SARS-CoV-2 virus encodes structural proteins required for the efficient formation of infectious virions; these include the spike (S), envelope (E), membrane (M) and nucleocapsid (N) proteins [7].

The key determinant of the host specificity of β-CoVs is the surface-located S protein, which plays critical roles in infection by mediating viral attachment to host cell-surface receptors and facilitating viral entry [8]. The S protein consists of two large regions: the N-terminal S1 and C-terminal S2 [9]. S1 is responsible for recognizing host-cell receptors, including the receptor-binding domain (RBD), and has higher sequence variability than S2 (S1 shares around 70% identity with that of other human β-CoVs). The membrane-embedded S2 region responsible for fusion is more highly conserved than that of S1 [8,9]. In SARS-CoV-2, the RBD in S1 allows the virus to bind directly to the peptidase domain of the host angiotensin-converting enzyme 2 (ACE2) complex, mediating virus entry into sensitive cells [10]. Notably, compared to SARS-CoV, SARS-CoV-2 has a higher binding affinity to ACE2 (which is the common receptor for both SARS-CoV-2 and SARS-CoV), with a broader interaction with ACE2 (suggested due to dynamics-based correlated motions and the electrostatic energy perturbations) expressed not only in the lungs but also in the kidneys, testes and heart [10,11,12,13,14].

Mutations in the genome sequence of SARS-CoV-2 are responsible for the emergence of new SARS-CoV-2 variants, many of which are characterized by higher transmission rates [15,16,17].

Recently, we conducted an analysis and identified for the first time viral prion-like domains (PrDs), which we suggest are novel regulators of virion assembly with a role to play in virus-host cell interactions [18,19]. These studies were in alignment with previous studies, showing that in addition to the pathological role prions play in humans—being implicated in Alzheimer’s and Parkinson’s diseases, diabetes, and many other human pathologies—protein misfolding also plays important physiological roles in eukaryotes and prokaryotes [20,21,22,23].

Though the detailed molecular mechanisms underlying prion formation remain elusive, asparagine (Q)- and glutamine (N)-rich regions characterized by altered hydrophobicity and net sequence charge are known to drive prion formation. This is the basis for a number of algorithms for identifying candidate prionogenic domains [24,25]. One such algorithm is prion-like amino acid composition (PLAAC) analysis, which allows for the evaluation of prion-like domains based on the hidden Markov model (HMM) that also incorporates other prion-like domain-predictive algorithms PAPA, DIANA, and FoldIndex [26,27,28].

Although structures for the variants of SARS-CoV-2 were extensively researched using CryoEM, modeling, molecular dynamics and other methods to study the impact on the binding affinity for each amino acid in contact with ACE2, the prionogenic properties of SARS-CoV-2 have not yet been studied [29,30,31,32].

In this study, we performed the first detailed evaluation of PrDs in the spike protein of SARS-CoV-2 and compared them to PrDs from other human-pathogenic β-CoVs. We also analyzed PrDs in the spike protein of the variants of concern (VOC) B.1.617.2 (Delta) and B.1.1.529 (Omicron), variants of interest (VOI) and variants being monitored (VBM), such as B.1.1.7 (Alpha), B.1.351 (Beta), P.1 (Gamma), B.1.427 (Epsilon), B.1.617.1 (Kappa) and P.2 (Zeta), some of which are known for their ability to escape antibody neutralization [33,34,35,36].

Further analyses of these PrD-containing proteins in SARS-CoV-2 may improve our understanding of the COVID-19 infection and provide new insights into its pathophysiology and novel targets for developing therapies, including antiprion compounds with binding properties to prion proteins.

## 2. Materials and Methods

### 2.1. Protein Sequences

To identify the PrDs present in viral proteomes, protein sequences were obtained from the UniProt Knowledge Base and National Center for Biotechnology Information (NCBI) database [37,38].

### 2.2. Identification of PrDs in Viral Proteomes

The presence of PrDs in β-CoV proteomes, found using the PLAAC algorithm, and the output probabilities for the PrDs were constructed based on amino-acid frequencies and similarities with PrDs in *Saccharomyces cerevisiae*. Using the parameter Alpha value (which is used to allow a continuous interpolation between the tested organism-specific background frequencies and *S. cerevisiae* background frequencies) of 1.0 we employed species-independent scanning to identify the PrDs in β-CoV.

We used a 3.0 log-likelihood ratio (LLR) cutoff, which reflects the maximum sum of per-residue log-likelihood ratios for any subsequence of length L_core_ that falls partially or entirely within the prion-like domain state in the HMM Viterbi parse within a provided sequence [24]. Prion-like domain amino-acid positions were determined based on the PLAAC algorithm program analysis.

### 2.3. Statistical Analysis

All statistical analyses were conducted using the Statistical Package for Windows (version 5.0) (StatSoft, Inc., Tulsa, OK, USA). Data were compared between viruses using an χ^2^ test or Fisher’s exact test. To detect differences in multiple comparisons, one-way analysis of variance (ANOVA) was fitted with the standard confidence interval of 95%. *p*-values < 0.05 were considered statistically significant.

## 3. Results

Using the prion-prediction PLAAC algorithm, we analyzed structural proteins derived from the UniProtKB and NCBI databases and identified PrDs in the S proteins of all β-CoVs analyzed in this study (Supplementary Figure S1). The LLR scores of PrDs of the S proteins were practically identical within the studied β-CoVs, ranging from 2.828 to 4.856 (Supplementary Figure S2). Notably, with more precise mapping of PrDs within these proteins, we found a striking difference in their localization, with SARS-CoV-2 being the only virus with PrDs identified within the RBD of the S protein (Table 1).

**Figure 1:**
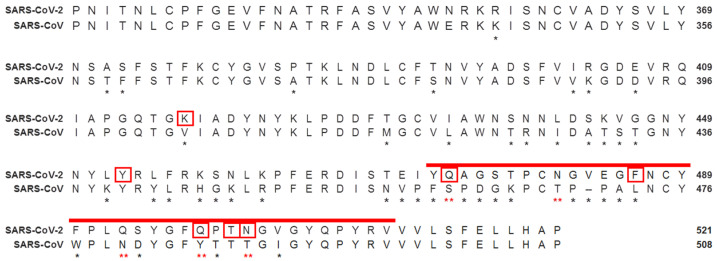
Analysis and comparison of mutations in the RBD of SARS-CoV-2 and SARS-CoV. The RBD of the SARS-CoV-2 (NCBI reference sequence: YP_009724390.1) spike protein was aligned with the closest related human βCoV, SARS-CoV (NCBI reference sequence: AYV99817.1). The PrDs of SARS-CoV-2 are red. Different residues are denoted by * beneath the consensus position. The amino acids asparagine (Q) and glutamine (N) in the PrDs of the SARS-CoV-2 RBD that differ from the amino acids in the SARS-CoV RBD are denoted by a red ** beneath the consensus position. Amino acids of the SARS-CoV-2 RBD that bind to ACE2 are marked with red boxes. RBD—receptor-binding domain.

Considering that although SARS-CoV-2 and SARS-CoV (which are the closest related human β-CoVs pathogens) share the same host-cell receptor ACE2, SARS-CoV-2 binds tighter to it; therefore, we hypothesized that the presence of PrDs in the RBD of the SARS-CoV-2 might explain this phenomenon [10]. Consistent with this hypothesis, we found that SARS-CoV-2, along with other residue substitutions, has five substituted amino acids in the RBD compared to SARS-CoV. The following are the substitutions in the RBD: S460→Q474, T488→N481, N480→Q493, Y485→Q498 and T488→N501, which form a hydrophobic Q/N-rich region that enables the prionogenity of the SARS-CoV-2 RBD (Figure 1).

**Figure 2:**
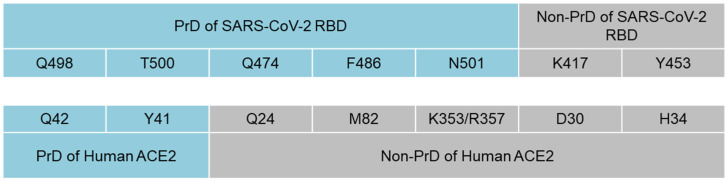
Interactions between amino acids of PrDs and non-prion-like regions of SARS-CoV-2 RBD and ACE2. Amino acids Q498 and T500 from the PrD of the SARS-CoV2 RBD interact with Y41 and Q42 within the PrD of ACE2, while Q474, F486 and N501 from the PrD of the SARS-CoV-2 RBD bind to Q24, M82 and K343 from the non-PrD of ACE2. K417 and Y453 were the only amino acids of the SARS-CoV-2 RBD that were outside the viral PrD and bound to ACE2.

We next analyzed the presence of prion-like domains in the ACE2 protein and found PrDs within the α1 helix of ACE2 (aa 40–65 and 93–106) (Supplementary Figure S3). Using the data from previous analysis which modulated the interface between the SARS-CoV-2 RBD and ACE2, we identified a pattern in which five of the seven amino acids that interact between the SARS-CoV-2 RBD and host cell ACE2 are localized within the PrDs of SARS-CoV-2 RBD, ACE2 or both of them (Figure 2) [39]. Thus, Q498 and T500 from the PrD of the SARS-CoV2 RBD interact with Q42 and Y41 within the PrD of ACE2. Meanwhile, Q474, F486 and N501 from the PrD of the SARS-CoV2 RBD bind to Q24, M82, K343 and R357, respectively, of a non-PrD of ACE2. Notably, K417 and Y453 were the only residues of the SARS-CoV-2 RBD that were outside the viral PrD and bound to a non-PrD of ACE2 (Figure 2).

We analyzed the particularities of the PrDs in the SARS-CoV-2 variants, some of which are known to have a substantially increased binding affinity due to mutations in the S protein, thus suggesting that they may have greater prion-forming potential. To this end, we analyzed the correlation between the LLR score of the PrD within the RBD of the S proteins of the VOC, VOI and VBM.

**Figure 3:**
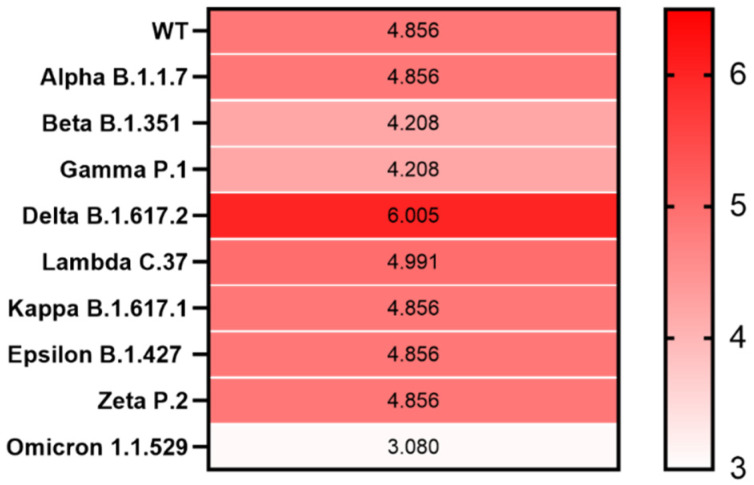
Heatmap showing PrD within the S protein in SARS-CoV-2 variants. The correlation between the LLR scores of the identified PrDs in the S protein across different SARS-CoV-2 variants is presented. Mean LLC scores of S protein are denoted using a color scale, ranging from white (minimum) to saturated red (maximum). Higher LLC scores indicate a higher possibility that the analyzed protein is a prion.

Compared with that of SARS-CoV-2 WT, we observed an elevated LLR score for the S protein from only the Delta (B.1.617.2) variant, with the LLR of 6.025, while from the emerging Omicron (B.1.1.529), the LLR was only 3.080 (Figure 3 and Supplementary Figure S4).

## 4. Discussion

This study is the most complete evaluation of PrDs in the S protein of SARS-CoV-2 variants. The results highlight some previously unknown and unique characteristics of SARS-CoV-2 that may play a role in the pathogenesis of COVID-19.

In this study, we used a high threshold of the PLAAC score for protein identification: only proteins with a high probability of prionogenic properties were included in the analysis. We found that although different β-CoV members contain PrDs in the S proteins, SARS-CoV-2 is the only member that has a PrD in the RBD of the S protein that binds to the ACE2 receptor employed for host-cell entry. Furthermore, we discovered specific amino acids (Q474, N481, Q493, Q498 and N501) that enable the prionogenity of the SARS-CoV-2 RBD that directly interacts within ACE2.

Inspecting the atomic interaction of SARS-CoV-2 and ACE2 showed that many pairwise interactions occur within the intrinsic disorder, which were detected as prion-like segments [13,14,24,33].

Notably, since five of the seven amino-acid interactions that occur between the RBD of SARS-CoV-2 and ACE2 are within their PrDs, it is also interesting to consider whether the prion-prion interaction between the virus and human receptor takes place in COVID-19, and whether it adds a special value for the higher affinity to their binding. Since other β-CoVs were shown to lack the PrDs in the RBD, this means that the presence of PrDs is beneficial, but not necessary, for receptor-mediated virion attachment to the host cell. One of the critical goals of our previous studies was to show that PrDs identified in viruses may have important functional roles to play in virulence and are particularly associated with viral adhesion and entry [18,19]. This study provides proof of this concept, demonstrating that the presence of PrDs in the RBD of SARS-CoV-2 enhances viral binding to its host receptor compared to that of SARS-CoV, which lacks PrDs in its RBD structure.

Furthermore, across all emerging SARS-CoV-2 variants, we observed the highest LLR scores in the S protein of the SARS-CoV-2 Delta (B.1.617.2) variant. This is notable since the Delta (B.1.617.2) variant is known for its highest transmissibility. The highest viral load is over 1000 times higher in people infected with the Delta variant than those infected with the original coronavirus strain, and the Delta variant is associated with higher mortality and greater risk of hospitalization [40]. However, the emerging Omicron (B.1.1.529) variant, although known for its high transmissibility, for now, appears to be milder than previous strains and has the lowest LLR among all SARS-CoV-2 variants. Further analyses of these PrD-containing proteins in SARS-CoV-2 may improve our understanding of the COVID-19 infection and provide new insights into its pathophysiology and novel targets for developing therapies.

## Figures and Tables

**Table 1:** Comparison of the distribution of PrDs within the S protein among different β-CoV human pathogens.

	S Protein		
	Domain	Prion-like Domain AA Position	LLR Score
SARS-CoV-2	RBD	473–510	4.856
SARS-CoV	HR1	900–910	4.426
MERS-CoV	NA	Non-detectable	4.49
HCoV-OC43	NA	Non-detectable	2.828

LLR: log-likelihood ratio.

## Data Availability

Not applicable.

## Supplementary Materials

The following supporting information can be downloaded at: https://www.mdpi.com/article/10.3390/microorganisms10020280/s1: Figure S1, Graphical representation of the LLR scores in the PrDs of S proteins from different β-CoVs; Figure S2, LLR scores showing the predicted putative PrDs in S proteins of β-CoVs; Figure S3, Graphical representation of the LLR scores of PrDs in the ACE2 protein; Figure S4, Graphical representation of the LLR scores of PrDs of the S proteins from different SARS-CoV-2 variants.
